# Parental measurement of height in growth hormone–treated children in the hospital setting proves valid: an observational study - potential for replacement of outpatient clinic visits to the home setting

**DOI:** 10.1007/s00431-023-05232-5

**Published:** 2023-10-16

**Authors:** Anouk J. W. Remmits, Ghislaine A. P. G. van Mastrigt, Silvia M. A. A. Evers, Hedi L. Claahsen-van der Grinten, Petra A. van Setten

**Affiliations:** 1 Department of Paediatric Endocrinology, Amalia Children’s Hospital, Radboud University Medical Centre, Nijmegen, The Netherlands; 2 School for Public Health and Primary Care, Department of Health Services Research, Faculty of Health, Medicine and Life Sciences, CAPHRI, Maastricht University, Maastricht, The Netherlands; 3 Netherlands Institute of Mental Health and Addiction, Trimbos Institute, Utrecht, The Netherlands

**Keywords:** Height, Validity, Portable stadiometer, Parentally reported, Transitioning outpatient visits to home care

## Abstract

Reliable height measurement plays a pivotal role in evaluating the efficacy of costly growth hormone (GH) therapy in children. Currently, regularly outpatient clinic visits are needed to accurately measure height. The outpatient clinic visits are time-consuming for parents as well for health care professionals. This observational study aimed to investigate the validity of parentally performed height measurements compared to height measurements in the outpatient setting. An observational study was performed at the outpatient clinic of Amalia’s Children’s Hospital Nijmegen. A portable stadiometer (PS) was developed for height measurements at home. Measurements with the PS were performed by the researcher (PSR) and parents/caregivers (PSP). Measurements performed with the electronic digital ruler (EDS) were considered as the gold standard. The parents were potentially unblinded for the gold standard measurement (EDS). Descriptive statistics, Wilcoxon signed-rank, and Pearson’s correlation tests were performed. The Bland–Altman plots were made to illustrate the correlation of the PSR or PSP with the gold standard. The correlation between the height measurements with PSR or PSP compared to the EDS was substantial (PSR: *r* = 0.9998, *R*^2^ = 0.9996, *P* < 0.001; PSP: *r* = 0.9998, *R*^2^ = 0.9995, *P* < 0.001). However, a statistically significant underestimation of the PSR and PSP was observed (*P* < 0.001). The mean difference of the PSR and PSP was respectively − 0.21 cm ± 0.52 SD and − 0.30 cm ± 0.62 SD in comparison to the EDS. The Bland–Altman plots illustrated that 95% of the PSR measurements were between − 1.03 and 0.60 cm and 95% of the PSP measurements were between − 1.26 and 0.66 cm compared to the EDS.

* Conclusion*: We found a strong correlation between the PSR or PSP and the EDS, with only a minor underestimation of approximately 0.2–0.3 cm. In our opinion, this underestimation is clinically irrelevant as it does not result in an adjustment in GH dose. To conclude, parental height measurements could be a promising tool as it partially replaces outpatient clinic visits needed for measurements of height. Further studies are required to confirm this statement.

**Table:** 

**What is Known:** *• The immense impact of the coronavirus disease 2019 (COVID-19) pandemic on health care has increased telemedicine worldwide. For adequate integration of telemedicine in paediatric growth hormone treatment, reliable height and weight measurements in the home setting are required.* *• Earlier studies have shown that parents are capable to reliable perform height measurements in healthy children.*	
**What is New:** *• To our knowledge, this is the first study to show a strong correlation between the height measurements with a portable stadiometer by parents and those made with the electronic digital ruler (gold standard) in children treated with growth hormone. There was only a minor underestimation of approximately 0.2–0.3 cm, which we anticipated to be clinically irrelevant.* *• Therefore, home height measurements can at least partly replace costly outpatient visits for children being treated with growth hormone as part of an uncomplicated course. Moreover, these results may also be promising for implementation in other paediatric populations besides children treated with growth hormone.*

**Supplementary Information:**

The online version contains supplementary material available at 10.1007/s00431-023-05232-5.

## Introduction

In the Netherlands, the prevalence of growth hormone (GH) treated children is approximately 8–10/10,000 children. This is in accordance with previous reports (1/4000–1/10,000) [1–4]. GH treatment is prescribed for several medical indications, including GH deficiency, Turner syndrome, and small for gestational age without catch-up growth [5]. The primary goal of this treatment is to promote height velocity and to improve final height. The starting dose is calculated according to body surface area (BSA) and depends on the specific indication. During follow-up, the GH dose is adapted based on auxological measurements (height and height velocity, weight and interval height increase) and serum insulin-like growth factor (IGF-1) concentrations. To this end, the paediatric endocrinologist needs accurate height measurements to adapt the GH dosage during follow-up visits every 3–4 months [6]. According to the WHO and Dutch guidelines for GH treatment, the gold standard for height measurements are respectively height boards and validated electronic digital stadiometers (EDS) which are not available in all specialised outpatient clinics [7].

The immense impact of the coronavirus disease 2019 (COVID-19) pandemic on healthcare has increased telemedicine worldwide [8]. Due to the pandemic, a significant number of physical outpatient clinic visits were replaced by online consultations via telemedicine [9]. It is likely that in the future, more telemedicine will be permanently integrated into clinical practice also for patients followed by paediatric endocrinologists [10]. Furthermore, earlier studies have shown that the use of telemedicine lowers the burden of disease for children and parents/caregivers and results in increased efficacy for physicians [11, 12]. In this context, also an adaptation of the follow-up of patients treated with GH by online consultations can be discussed.

However, as stated above, reliable height measurements at home are a prerequisite to performing visits via telemedicine for patients on GH treatment. Reports in the literature are rather scarce and inconsistent. Some articles show that parentally (self)reported height and weight measurements correlate fairly good to measurements by observers particularly when the parents were instructed [13, 14]. However, also under- and overestimation are reported [15–17]. These results indicate that caregivers are in potentially capable of performing height measurements at home in the context of partially replacing outpatient visits by telemedicine with home-based check-ups for GH treatment. However, appropriate tools for home height measurements are lacking. The general aim of this study was to investigate the validity of parentally measured height. Therefore, we firstly assessed the accuracy of a prototype stadiometer by comparing the height measured with the prototype by a trained researcher to the measurement of the height with the EDS. Secondly, we studied the validity of the parentally measured height, by comparing the height measurements of parents with the prototype stadiometer with those measured by the gold standard (EDS). When parents are able to provide reliable home measurement of their child’s height, home-based check-ups can at least partially replace outpatient clinic visits for children being treated with GH.

## Patients and methods

### Patients

For this observational study conducted at the Amalia’s Children’s Hospital Nijmegen, children were treated with GH, aged between 4 and 18 years old, and able to speak Dutch were included. Children with scoliosis, limb length differences, and inability to measure height in a prone position were excluded from this study. During the period May–July 2021, children were recruited by the researcher (AR) during their outpatient clinic visit to their paediatric endocrinologist.

### Power calculation

Based on the power calculation for Wilcoxon signed-rank test (power 80%, *α* 0.05), an estimation of a minimum of 36 children was determined as sample size for this study.

### Devices to measure height

For this study, we used the following two devices:

1. Electronic digital stadiometer (EDS): The EDS is routinely available at the outpatient clinic and is used as the gold standard to measure height in children (IDC 250 DW from Prior Medical Systems).
2. Portable stadiometer (PS): This PS prototype was developed in collaboration with a company specialised in customised industrial innovations (Tentije Industrial Automation).

**Fig. 1:**
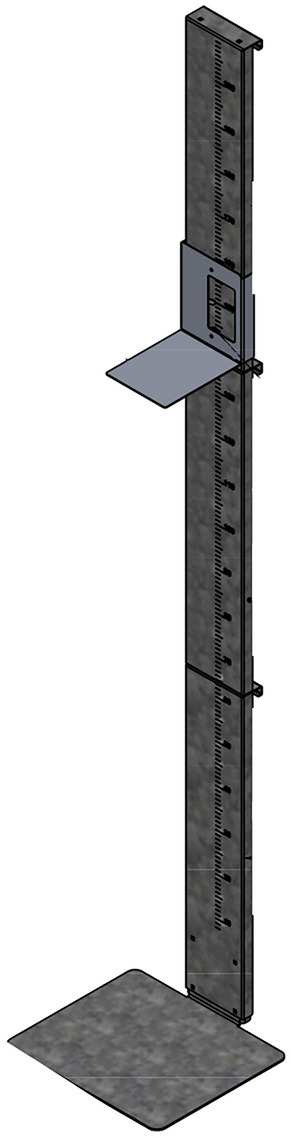
Conceptual design of portable stadiometer

The PS, built of high-quality materials (stainless steel), was for this reason stable and easy to install with a robust platform on a smooth surface and movable headboard and did not need to be attached to the wall. The measuring range graduation in centimetres and millimetres was 50–200 cm and 50 mm, respectively (Fig. 1). The cost of one stadiometer was approximately 50 euros. Overall, the stadiometer developed was comparable to commercially available stadiometers.

### Height measurements

**Fig. 2:**
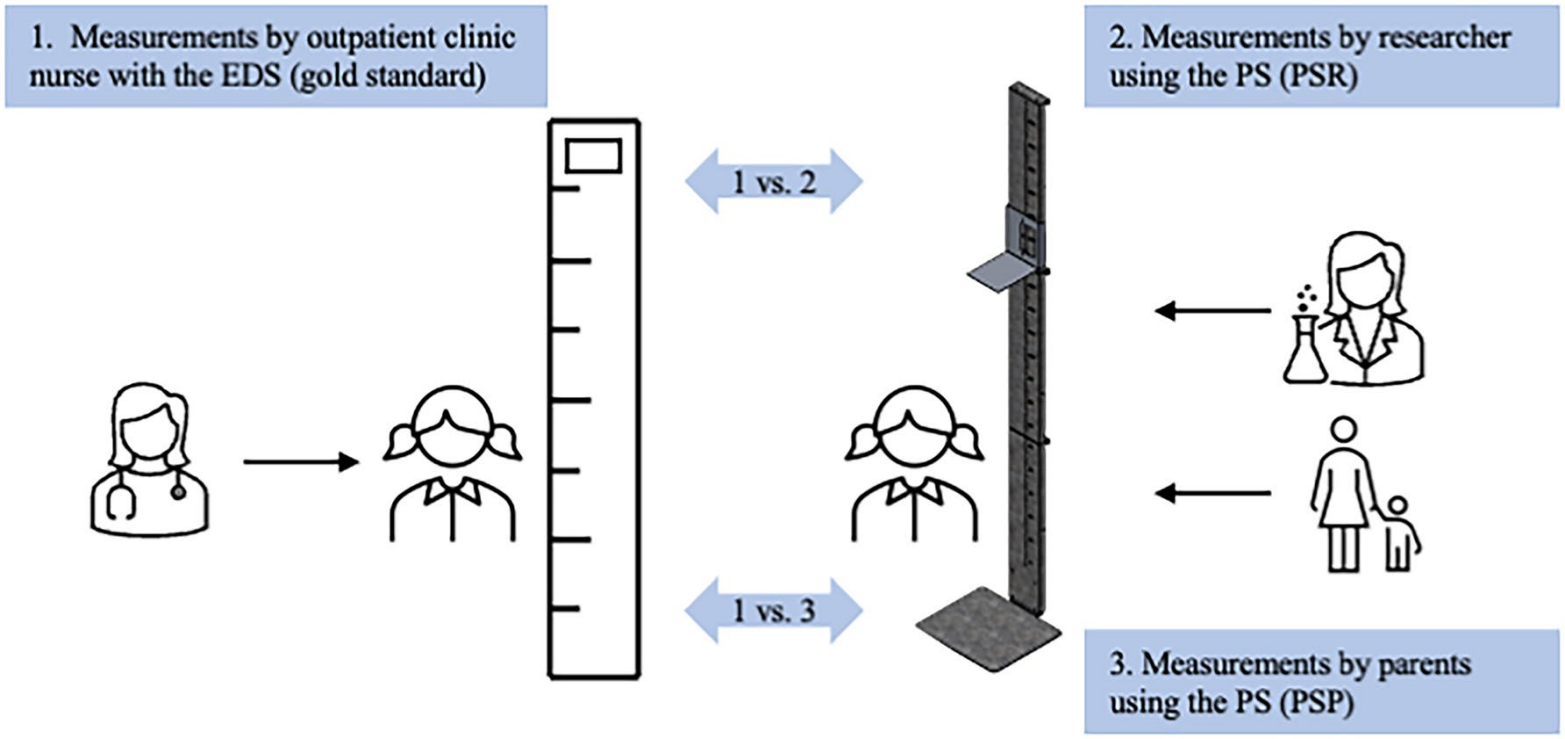
Conceptual design of the study. EDS, electronic digital stadiometer; PS, portable stadiometer; PSR, portable stadiometer measured by researcher; PSP, portable stadiometer measured by parents/caregivers

All patients were measured three times during the same regular outpatient clinic visit (Fig. 2). The following measurements were performed:

1. Height measurement with electronic digital stadiometer (EDS) by an outpatient clinic nurse
2. Height measurement with portable stadiometer (PS) by the researcher (PSR; AR)
3. Height measurement with portable stadiometer (PS) by parents/caregivers (PSP)

The children were first measured with the EDS (the gold standard once). Subsequently, the researcher explained the protocolled PS measurement method to the participants and parents (see procedure) [7]. The participants were measured twice with PSR and thereafter twice by PSP; parents/caregivers were instructed to do the height measurements as they would perform at home following the instructions they received. The researcher observed the parents during the measurement of their child but did not correct them. The researcher was blinded for the measurement with the EDS. Parents were potentially not blinded, because they present during the outpatient clinic visit during which the child was measured with the EDS by the outpatient clinic nurse.

### Procedure

1. Measure the length without shoes or slippers; barefoot.
2. The head in upright position.
3. Put the patient against the ruler with the heels together, heels against the wall and upright position.
4. Gently lower the distance clamp to the head.
5. Extend the child’s neck as far as possible by keeping the head straight up.
6. The child should stay in the same position (with the heels together, heels against the wall and upright position.

### Data collection

All data were stored in Castor EDC. The company had no role in the study design, execution, analysis, interpretation of the data, or the decision to submit manuscript. We followed the Strengthening the Reporting of Observational Studies in Epidemiology (STROBE) checklist for reporting [18].

### Data analysis

The mean of two measurements was calculated for PSR and PSP measurements and used for the data analysis. To validate the PS, the measurements performed by the researcher and parents/caregivers were independently compared with the measurements performed with the EDS. GraphPad version 9.1.2 was utilised for the statistical analysis. Descriptive statistics (mean, standard deviation, and range) were performed for the analysis of baseline characteristics (age, gender, and medical condition behind the GH treatment). The differences between the height measurements with the PSR or PSP and the EDS were calculated by subtracting respectively the PSR and PSP values from the EDS. Thus, positive differences implied that the measurements with the PS had a higher value than the measurement performed with the EDS. To assess whether the measurements of the PSR and PSP were significantly different when compared to the measurements made with the EDS, a Wilcoxon signed-rank test was performed. A Pearson’s correlation test was performed to assess the correlation between PSR or PSP and EDS. Furthermore, a Bland–Altman plot was designed to investigate the level of agreement between the PSR/PSP and EDS.

### Ethical considerations

Children and parents were informed about the study’s goals and procedures; thereafter, they were asked for written informed consent to collect and analyse data. The procedures followed were in accordance with the World Medical Association Declaration of Helsinki. The Medical Research Ethics Committee of Nijmegen determined that this study did not fall within the remit of the Dutch “Medical Research Involving Human Subjects Act” (No. 2021–7506). Written informed consent was obtained from patients 12 years and older and from parents/caregivers of children younger than 16 years.

## Results

### Baseline characteristics

A total of 64 children participated in this study. One participant has withdrawn his consent, resulting in 63 participants (35 females/28 males) available for the analysis with varying GH indications. The mean age of the children who participated in this study was 10.51 (range 4–17 years). The baseline characteristics of the participants in this study are described in Table 1.

**Table 1:** Characteristics of children participating in this study

	**Children (*N* = 63)**
**Age (SD)**	10.51 (3.51)
**Gender, *n* (%)**	
Female	35 (55.6)
Male	28 (44.4)
**Indication for GH treatment, *n* (%)**	
Syndromes associated with short stature	11 (17.5%)
Turner syndrome	9
Noonan-syndrome	2
Skeletal dysplasia	5 (7.9%)
SHOX-gene mutation	4
ACAN-mutation	1
SGA	19 (30.2%)
SGA (without catch-up growth)	16
SGA (Silver-Russel syndrome)	3
Endocrine disorders	28 (44.4%)
Isolated GH deficiency	16
GH deficiency with additional deficiencies (following oncological treatment)	7
Panhypopituitarism	5

Characteristics of the children participating in this study

*GH* growth hormone, *SD* standard deviation, *ACAN-mutation* mutation in the aggrecan gene, *SGA* small for gestational age, *SHOX* short-stature homeobox

### Comparison of height measurement between the EDS and PS

**Fig. 3:**
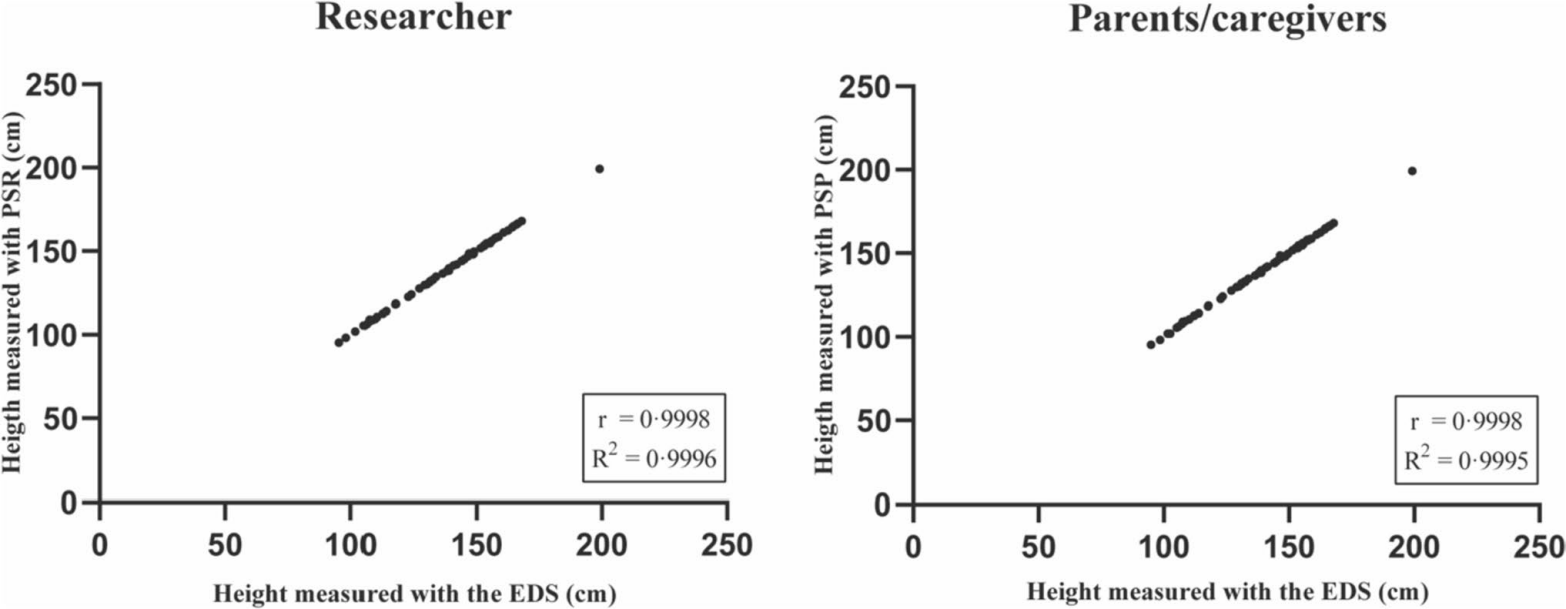
Pearson’s correlation between height measured with an EDS and the PSR or PSP. Correlation between the height measured with the EDS (in cm) and the PSR or PSP (in cm). Correlation between measurement made with the PSR (left) and with the PSP (right). n = 63. EDS, electronic digital stadiometer; PSR, portable stadiometer measured by researcher; PSP, portable stadiometer measured by parents/caregivers

The Pearson’s correlation coefficient illustrated a strong correlation (PSR: *r* = 0.9998, *R*^2^ = 0.9996, *P* < 0.001; PSP: *r* = 0.9998, *R*^2^ = 0.9995, *P* < 0.001) between the EDS and the PSR/PSP (Fig. 3). The mean height ± standard deviation (SD) measured with the EDS was 138.1 ± 21.8. The mean difference ± SD between the PSR and the EDS was − 0.21 cm ± 0.52, demonstrating that the height measured with the PSR slightly underestimated the height measured with the EDS. The height difference measured with the PSR was 0.15%. of the mean measured with the EDS. The PSP also underestimated the child’s height, the mean difference of the PSP compared to the EDS was − 0.30 cm ± 0.62, which was 0.21% of the mean measured with the EDS. The Wilcoxon signed-rank test revealed that the underestimation of the height (both PSP and PSR) was statistically different from the measurement made with the EDS (*P* < 0.001) (Table 2).

**Table 2:** Height measured with an EDS compared to the PSR and PSP

	**Mean height measured** **(cm)**	**SD of measured height**	**Difference with EDS (cm)**	**SD of difference with EDS (TEM)**	**P-value for difference in mean**
EDS	138.1	21.82	/	/	/
PSR	137.9	21.87	-0.21	0.41	*P* < 0.001
PSP	137.8	21.92	-0.30	0.49	*P* < 0.001

*EDS* electronic stadiometer, *PSR* portable stadiometer measured by researcher, *PSP* portable stadiometer measured by parents/caregivers, *TEM* technical error of measurement

### Bland–Altman plots for agreement between EDS and PS measurements

**Fig. 4:**
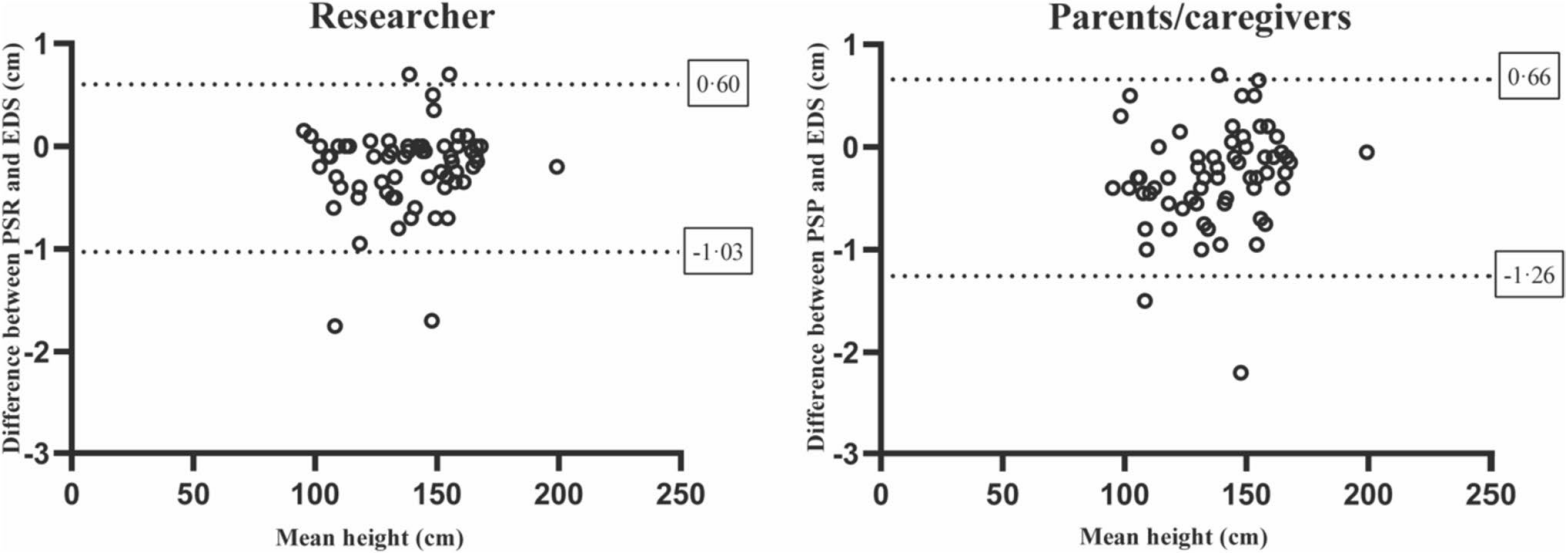
Bland–Altman plots of the difference between the EDS and the PSR or PSP. The difference between the mean height of the EDS and PSR or PSP (X-axis) and the difference with the PSR or PSP (Y-axes) is plotted for the PSR (left) and the PSP (right). The dotted lines represent the upper limit of agreement (mean + 1.96SD) and lower limit of agreement (mean – 1.96SD). n = 63. EDS, electronic digital stadiometer; PSR, portable stadiometer measured by researcher; PSP, portable stadiometer measured by parents/caregivers

The Bland–Altman plots illustrate the agreement between the PSR/PSP and the EDS on an individual level (Fig. 4). The measurements of the PSR showed that the researcher measured 95% of the children between − 1.03 and 0.60 cm from the height measured with the EDS. In addition, 95% of the measurements of the PSP were between − 1.26 and 0.66 cm from the height measured with the EDS.

## Discussion

We found a strong correlation between height measurements performed with the PSR and PSP compared to the EDS in the hospital setting. Only a minor underestimation with a small, likely clinically irrelevant difference in the mean height was found between both PS groups and EDS. Reliable height measurements by parents/caregivers in the hospital are essential to investigate in the future whether height measurements at home can be performed reliably. Literature about the validity of home height measurements is scarce and rather inconsistent. However, there are several studies on the validity of self-reported height measurement in children [13, 14, 19, 20] showing a fairly good relationship between the parentally reported measurements by using several devices and those measured by observers with a tendency towards a slight underestimation of the height of the child. There are also reports showing inaccuracy. The observation of a good correlation with a slight underestimation by the parents is in accordance with our results. Carsley et al. showed that the technical error of measurement (TEM; SD of the difference) between trained and non-trained observers (inter-observability) was 0.45 cm [21]. This is comparable to our TEM (0.41 the PSR and 0.49 for the PSP). In our study, the parents/caregivers and the researcher both underestimated the height of the children by 0.30 cm (0.21% of the mean) and 0.21 cm (0.15% of the mean), respectively. On an individual level, the 95% of the differences between the EDS and PSP measurements are expected to be between -1.26 and 0.66 cm). The same was found for the researcher (between -1.03 and 0.60 cm). As such, the observed differences are a minor underestimation of the length with the PS. This minor underestimation may be due to the robustness of the non-electronic PS. We anticipate that the observed small underestimation has neither clinical significance nor implication for GH dosing. To estimate the margins of error for our study, we used the formula of Mosteller and the growth data of the National Dutch Growth Diagrams published by TNO [22–24]. We calculated the error of measurement which was needed to increase the GH dose by 0.1 mg/dg/subcutaneous (sc) for both sexes and for different dosages. We found that the maximum error of measurement varied between a minimum of 2.9 cm (girls and a GH dose of 1.4 mg/m^2^/dg/sc) and a maximum of 7.9 cm (boys and a GH dose of 0.7 mg/m2/dg/sc) (data not shown). As such, we concluded that the observed small underestimation did not result in a different dosing advice of GH in the individual patient. The difference in measurement between the EDS and PSR/PSP may be due the fact that the ESD was measured by the outpatient clinic nurse and the PSR by the researcher and the fact that the researcher instructed the parents/caregivers.

The PS, showing a good correlation and only a minor underestimation in the hospital setting, may be a sufficient tool for height measurements at home and as such for home-based check-ups for monitoring GH treatment. Earlier studies have shown that these online consultations may hold potential benefit for both healthcare providers and children [11]. Whereas we believe that home-based check-ups are suitable for most children with uncomplicated GH treatment, this may not be appropriate for those children with multiple pituitary hormone deficiencies and those with compliance issues or poor growth response. Obviously, in these patients and whenever physical examination is necessary, outpatient clinic visits remain essential.

This is the first study to examine the validity of height measurements with a simplified prototype, in the context of partially transferring outpatient clinic visits to home-based check-ups for children being treated with a growth hormone. A substantial strength of this study was the inclusion of height measurements performed by both parent/caregivers and by a researcher who was already experienced in performing height measurements. Furthermore, the mean of the measured values was taken to increase the accuracy of the outcomes. A limitation of this study was that we did not blind the parent/caregivers; the majority knew the height of their child as measured by the EDS before the measurement was made with the PS because they were present during the height measurement with the EDS. We assume that the likelihood of this bias is limited because the researcher observed the parents/caregivers during the procedure but did not correct them. In addition, this study focused only on validating home height measurements and did not consider weight measurements. Weight and height measurements are both important for home-based check-ups.

In the future, we anticipate a pilot validation study with the portable stadiometer in the home setting of the child. Huybrechts et al. illustrated the importance of proper instructions and motivation for home height (and weight) measurements. As such, we will provide appealing instructions for both children and parents/caregivers [25]. Furthermore, it is worthwhile to test the user experience of both the children themselves and parent/caregiver.

In conclusion, we found that parental measurements of height proved valid. As such, parental measuring is a promising tool for partially replacing outpatient clinic visits to the home setting. Further studies are necessary to confirm our findings in the home setting.

### Supplementary Information

Below is the link to the electronic supplementary material.

Supplementary file1 (PDF 113 KB)

## Data Availability

The raw data that support the findings of this study are available on request from the corresponding author. The data will not be publicly available due to restrictions (their containing information that could compromise the privacy of research participants).
